# Isolation and Functional Characterization of Bidirectional Promoters in Rice

**DOI:** 10.3389/fpls.2016.00766

**Published:** 2016-05-31

**Authors:** Rui Wang, Yan Yan, Menglin Zhu, Mei Yang, Fei Zhou, Hao Chen, Yongjun Lin

**Affiliations:** ^1^National Key Laboratory of Crop Genetic Improvement and National Centre of Plant Gene Research, Huazhong Agricultural UniversityWuhan, China; ^2^Chinese Academy of Tropical Agricultural SciencesHainan, China

**Keywords:** rice, bidirectional promoter, stable transformation, GUS assay, GFP assay, deletion analysis, conservation analysis

## Abstract

Bidirectional promoters, which show great application potential in genetic improvement of plants, have aroused great research interest recently. However, most bidirectional promoters were cloned individually in the studies of single genes. Here, we initiatively combined RNA-seq data and cDNA microarray data to discover the potential bidirectional promoters in rice genome. Based on the expression level and correlation of each adjacent and oppositely transcribed gene pair, we selected four candidate gene pairs. Then, the intergenic region between each pair was isolated and cloned into a dual reporter vector pDX2181 for functional identification. GUS and GFP assays of the transgenic plants indicated that all the intergenic regions showed bidirectional expression activity in various tissues. Through 5′ and 3′ deletion analysis on one of the above bidirectional promoters, we identified the enhancing region which sharply increased its bidirectional expression efficiency and the essential regions respectively responsible for its 5′ and 3′ basic expression activity. The bidirectional arrangement of the four gene pairs in six gramineous plants was also analyzed, showing the conserved characteristics of the four bidirectional promoters identified in our study. In addition, two novel *cis*-sequences conserved in the four bidirectional promoters were discovered by bioinformatic identification. Our study proposes a feasible method for selecting, cloning, and functionally identifying bidirectional promoters as well as for discovering their bidirectional regulatory regions and conserved sequences in rice.

## Introduction

Plant architecture, development, and interaction with environment are controlled by the expression of a series of genes (Chen F. et al., 2010; Li C. et al., 2011; Zhu et al., 2011). As a critical regulator of gene expression, promoters are important in plant biotechnology and functional genomics research for their great application potential in genetic engineering and theoretical significance in the exploration of transcriptional regulation mechanism (Cai et al., 2007; Yi et al., 2011; Walcher and Nemhauser, 2012; Ye et al., 2012; Balasubramani et al., 2014). Many researches have been focused on the cloning and analysis of unidirectional promoters, such as constitutive promoters, spatiotemporal promoters, and inducible promoters (McElroy et al., 1990; Pan et al., 2015; Vijayan et al., 2015). Bidirectional promoters, which generally refer to the intergenic region between two adjacent genes transcribed in opposite directions, show better applicability than unidirectional promoters in genetic improvement (Trinklein et al., 2004; Mitra et al., 2009; Yang et al., 2013). That is because a bidirectional promoter can drive the expression of two genes simultaneously, and thus can be time-saving in constructing expression vectors and pyramiding of multiple genes (Kumar et al., 2015). Besides, it is very critical for transgenic breeding to enable the functionally related genes to express in the same pattern in the receptors (Ha et al., 2010; Ogo et al., 2013). However, the unidirectional promoters with the same specific expression patterns are only available in limited quantities, and repetitious use of the promoters may have a negative impact on the stability and expression of transgenes (Peremarti et al., 2010). According to previous reports, the expression patterns of bidirectional promoters in opposite directions are similar in many cases due to the co-expression of the adjacent genes (Huang et al., 2007; Wang et al., 2009; Chen W. et al., 2010; Didych et al., 2013). Therefore, bidirectional promoters could also compensate for the lack of unidirectional promoters with the same expression pattern.

Much work has been done to analyze the bidirectional promoters in mammalian genome with experimental and bioinformatic methods (Trinklein et al., 2004; Yang and Elnitski, 2008; Uwanogho et al., 2010). The results suggest that the divergent gene pairs regulated by bidirectional promoters exhibit the characteristics of conserved arrangement, coexpression, and functional association (Xu et al., 2012; Didych et al., 2013; Meersseman et al., 2014; Yang and Elnitski, 2014). Since the sequences of promoters are known to be variable (Müller et al., 2007), for discovering bidirectional promoters, it is particularly helpful to investigate the characteristics of the divergent gene pairs regulated by them.

Bidirectional promoters have become a research focus in plants in recent years. With the development of plant genome sequencing, bioinformatic analyses in plants like rice, *Arabidopsis* and *Populus* have revealed that the divergent gene pairs regulated by bidirectional promoters have similar characteristics, such as coexpression, functional association, and conserved arrangement (Krom and Ramakrishna, 2008; Dhadi et al., 2009; Wang et al., 2009; Chen W. et al., 2010). The structural characteristics of bidirectional promoters in plants are similar to those in mammals, such as higher GC content and less TATA boxes (Dhadi et al., 2009). Besides, bidirectional promoters have been cloned in many species. In *Arabidopsis*, the tissue-specific and light-inducible bidirectional promoter between *cab1* and *cab2*, the tissue-specific bidirectional promoter between *At5g06290* and *At5g06280*, and the tissue-specific and stress-inducible bidirectional promoter between *At4g35985* and *At4g35987* have been cloned successively (Bondino and Valle, 2009; Mitra et al., 2009; Banerjee et al., 2013). All of these promoters can be widely used in gene functional analysis in *Arabidopsis*. Several bidirectional promoters from other species such as melon and *Capsicum annuum*, have been also cloned gradually (Shin et al., 2003; Wang et al., 2008). In rice, a few promoters have been found to show bidirectional expression activities (Huang et al., 2007; Singh et al., 2009; Dhadi et al., 2013). So far, there has been no report about the cloning and identification of bidirectional promoters using two reporter genes simultaneously with stable transformation in rice.

Rice is one of the most important food crops in the world and a model plant for functional genomic researches in cereals (Zhang, 2007). Therefore, it is highly necessary to introduce multiple genes into rice for genetic improvement (Ha et al., 2010; Yang et al., 2011; Ogo et al., 2013). Accordingly, discovery of bidirectional promoters in rice is very critical. Besides, more complete genomic information (Goff et al., 2002; Yu et al., 2002; Pan et al., 2014) and more explicit gene expression information (Wang et al., 2010; Kawahara et al., 2013; Sakai et al., 2013) will greatly facilitate the development of a high-throughput method for discovering bidirectional promoters.

Most of the known bidirectional promoters were found during the researches of single genes. In this study, we reported a method of selection, cloning, functional identification, and deletion analysis of bidirectional promoters for their *de novo* discovery in rice. We first selected four adjacent and oppositely transcribed gene pairs based on their expression levels and correlations, which were derived from the RNA-seq data of the Michigan State University Rice Genome Annotation Project Database (MSU), the Rice Annotation Project Database (RAP), and the microarray data of the rice cDNA microarray database (CREP). Subsequently, the intergenic regions between the four gene pairs were cloned for functional identification. GUS and GFP assays of the transgenic plants indicated that all the intergenic regions showed bidirectional expression activity in various tissues. With 5′ and 3′ deletion analysis of one bidirectional promoter above, we found the regulatory region responsible for its bidirectional expression activity. Meanwhile, the bidirectional arrangement of the four gene pairs in six gramineous plants showed the conserved characteristics of the four bidirectional promoters identified in our study. Then, we discovered two *cis*-sequences conserved in the four bidirectional promoters with MEME. The two *cis*-sequences showed overrepresentation in the intergenic regions between divergent gene pairs in rice genome under the reference of random promoters. Our study proposes a feasible method for selecting, cloning, and functionally identifying bidirectional promoters as well as for the discovery of their bidirectional expression regulatory regions and conserved sequences in rice.

## Methods

### Selection of candidate bidirectional promoters

RNA-seq data were obtained from the Michigan State University Rice Genome Annotation Project Database (MSU) and the Rice Annotation Project Database (RAP; Kawahara et al., 2013; Sakai et al., 2013), and the microarray data were downloaded from the rice cDNA microarray database (CREP; Wang et al., 2010). Based on the expression characteristics, the criteria for candidate divergent gene pairs regulated by bidirectional promoters in our study were set as: the maximum expression value of the gene pair was simultaneously higher than 10 in RNA-seq data and higher than 5000 in microarray data, and the expression correlation coefficient between the gene pair from RNA-seq data of 95 samples was higher than 0.4. Because only 36 samples were available in microarray data, which were not sufficient for reliable correlation analysis, we did not consider the correlation coefficient of the data from microarray data. According to the criteria, we chose four divergent gene pairs (Table 1) and isolated their intergenic regions (here designated as *BIP1, BIP2, BIP3*, and *BIP4*, respectively) for functional identification.

**Table 1:** **Four divergent gene pairs chosen for functional identification**.

**Intergenic regions**	**5′ gene**	**Minimum value**	**Maximum value**	**Mean value**	**3′ gene**	**Minimum value**	**Maximum value**	**Mean value**	**Pearson correlation coefficient**	**Spearman correlation coefficient**	**Database**
*BIP1*	LOC_Os02g42314	0.6	21.57	7.88	LOC_Os02g42320	0	31.16	11	0.67	0.62	MSU
		1.65	35.33	10.35		0	10.25	3.8	0.58	0.51	RAP
		2143.75	9118.85	5662.15		6009.55	15236.5	9940.64			CREP
*BIP2*	LOC_Os05g27940	1.58	88.59	17.19	LOC_Os05g27950	0.38	40.61	8.91	0.45	0.5	MSU
		2472.2	22698.9	13559.4		1381.8	7987.6	4900.6			CREP
*BIP3*	LOC_Os02g47000	0	30.11	13.56	LOC_Os02g47010	0.37	16.17	4.96	0.51	0.49	MSU
		0.05	639.27	19.56		0.23	720.79	45.81	0.84	0.93	RAP
		568.4	4000.9	1680.54		709.45	7154.35	3080.98			CREP
*BIP4*	LOC_Os03g22880	0	69.12	9.42	LOC_Os03g22890	0	184.71	15.17	0.65	0.78	MSU
		0.16	21.51	3.8		1.31	142.75	12.44	0.74	0.81	RAP
		274.55	13458.05	4949.52		1065.65	14857.15	7410.79			CREP

### Isolation and vector construction of *BIP1, BIP2, BIP3*, and *BIP4*

The genomic DNA of Minghui 63 (*Oryza sativa* L ssp. *indica*) was used as template to amplify *BIP1, BIP2, BIP3*, and *BIP4* with specific primers (Table 2). The PCR-generated fragments were respectively inserted into T-vector (Promega) and confirmed by sequencing with primers SP6 and T7. The sequence-confirmed clone containing *BIP1*/*BIP2*/*BIP3*/*BIP4* was digested by *Pst* I/*Bam*H I/*Bam*H I/*Bam*H I and was respectively cloned into a dual reporter vector pDX2181 (Ye et al., 2012).

**Table 2:** **Polymerase chain reaction (PCR) primers used in this study**.

**Primer name**	**Primer sequence (5′–3′)^a^**	**Purpose**
BIP1-F	AACTGCAGCTGGTCTCCTCTCTACTGTTG	Promoter clone
BIP1-R	AACTGCAGAGCTGCAAACATAACAAATATACC	Promoter clone
BIP2-F	CGGGATCCCTTGTGATAACCCTGTAGTG	Promoter clone
BIP2-R	CGGGATCCCTCTTCCTGAAGAAACCATC	Promoter clone
BIP3-F	CGGGATCCCTCGCTGAGCTACCAATAACC	Promoter clone
BIP3-R	CGGGATCCCTACACCACACCCACACCCCATT	Promoter clone
BIP4-F	CGGGATCCCTCGCCGGCGGCGTCGGC	Promoter clone
BIP4-R	CGGGATCCCGCAGAGGATTTTTCTTCTTC	Promoter clone
BIP1-2F	AACTGCAGCAGCTCGCAGCTCCCCT	5′ deletion analysis
BIP1-2R	AACTGCAGGCTGCAAACGAAATCGCCAC	3′ deletion analysis
BIP1-3R	AACTGCAGGGCCGCCGACGCGCAGGCCTA	3′ deletion analysis

a *The underlined letters indicate the restriction enzyme sites*.

### *Agrobacterium*-mediated rice transformation

The sequence-confirmed clones were transformed into the *Agrobacterium tumefaciens* strain *EHA105* by electroporation. Subsequently, all the constructs were introduced into Zhonghua11 (*O. sativa* L. ssp. *japonica*) by *Agrobacterium*-mediated transformation. The callus culture and transformation procedures were carried out as previously described (Hiei et al., 1994).

### Histochemical and fluorometric analysis of GUS activity

Histochemical staining of GUS activity in rice tissues was conducted essentially as previously described (Jefferson et al., 1987). Various tissues of T_0_ transgenic-positive transformants (root, leaf, sheath, panicle, stem, and mature seed) were incubated in GUS staining solution (50 mM sodium phosphate at pH 7.0, 10 mM Na_2_-EDTA, 0.1% Triton X-100, 1 mg/mL X-Gluc, 100 μg/ml chloramphenicol, 1 mM potassium ferricyanide, 1 mM potassium ferrocyanide and 20% methanol) at 37°C for 2–10 h after 15-min vacuum filtration. After GUS staining, the samples were incubated in 70% ethanol to remove chlorophyll and photographs were taken under a dissecting microscope (Leica MZFLIII).

Quantitative analysis of GUS activity was conducted as previously described (Xu et al., 2010). The total protein concentration in the supernatant was quantified using the Bradford assay (Bradford, 1976). GUS protein in the supernatant was determined fluorometrically with an INFINITE 200 photometer (Tecan Austria Gmbh, Ltd, Grodig, Austria). GUS activity was determined fluorometrically by measuring the amount of 4-methylumbelliferone (Mu) produced under the catalysis of GUS in 1 mg of total protein per minute. Five biological replicates were assayed for each construct. Ten transgenic lines were randomly divided into five groups and two transgenic lines were considered as one biological replicate.

### Histological and quantitative analysis of GFP

Histological analysis of GFP in rice tissues was detected and photographed under fluorescence microscope. Various tissues of T_0_ transgenic-positive transformants (root, leaf, sheath, panicle, stem, and mature seed) were sampled and observed under a fluorescence microscope (Leica MZ16F) using GFP filter sets and Leica Application Suite software.

The relative expression levels of *GFP* in rice tissues were detected by quantitative real-time PCR (qRT-PCR). Total RNAs of different rice tissues were extracted and reverse-transcribed as described previously (Wang et al., 2015a), and qRT-PCR was performed according to the same reference. The primers of *GFP* were GFP-F: 5′-ATCCGCCACAAC ATCGAGGA-3′ and GFP-R: 5′-TCGTCCATG CCGAGAGTGAT-3′, and the primers of *GAPDH* (the endogenous control) were GAPDH-F: 5′-CTGCAACTCAGA AGACCGTTG-3′ and GAPDH-R: 5′-CCTGTT GTCACCCTGGAAGTC-3′. Relative expression levels were determined using 2^−ΔΔC_T_^ method (Livak and Schmittgen, 2001).

### Melatonin treatment

Melatonin (*N*-acetyl-5-methoxytryptamine), which is known as an indispensable hormone related to many physiological activities in animals, has also been identified as an important signaling molecule in response to many stresses in plants. In order to test the response of the four bidirectional promoters to melatonin, melatonin treatment was performed on the transgenic plants according to the procedure described by Shi and Chan (2014).

### 5′ and 3′ deletion analysis of *BIP1*

**Figure 1:**
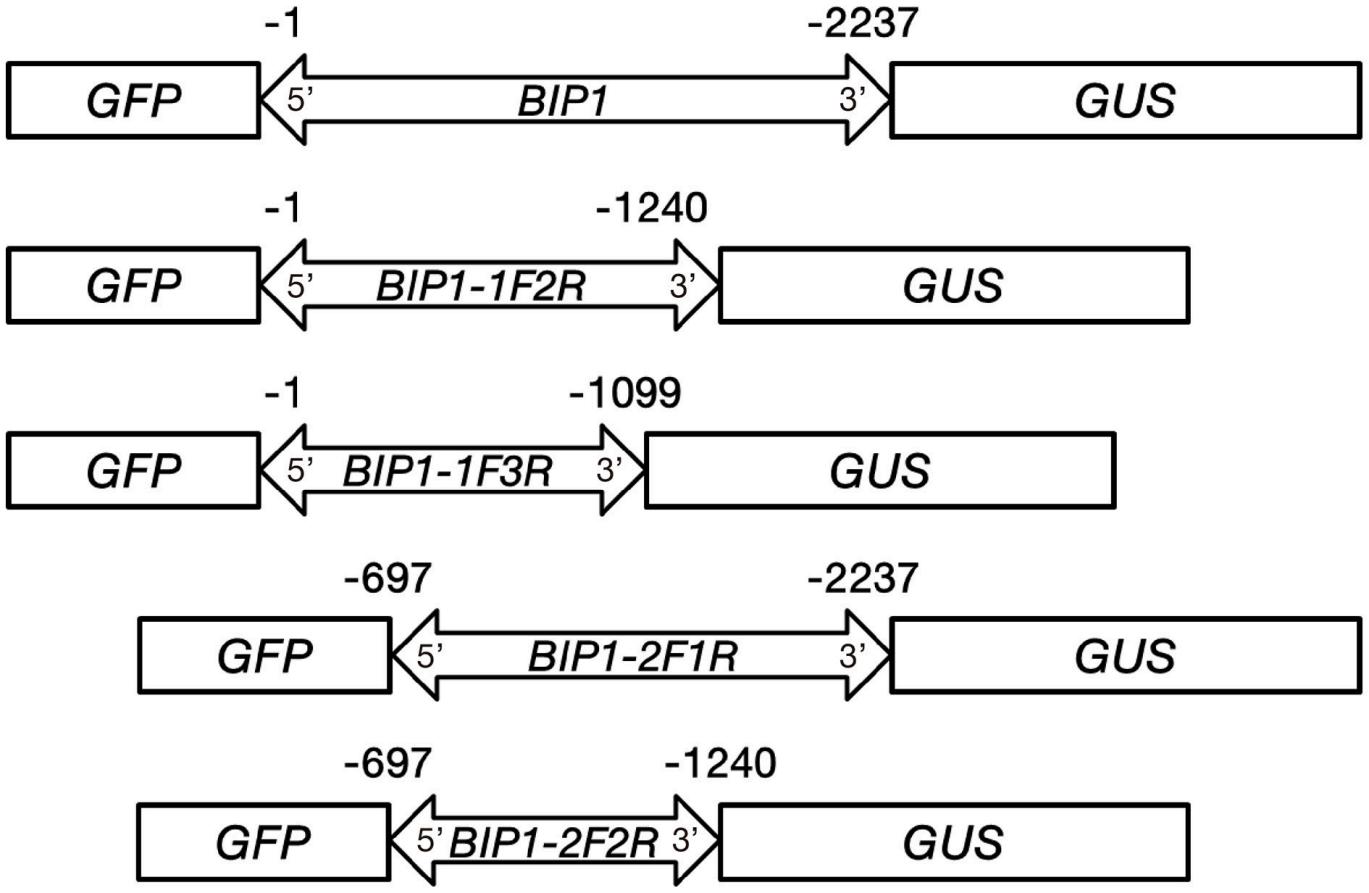
**Schemes of constructs carrying *BIP1* and different deleted versions fused with *GFP* and *GUS* reporter genes**.

Among the four bidirectional promoters above, *BIP1* showed the highest expression efficiency in both 5′ and 3′ orientations. Therefore, it was selected for 5′ and 3′ deletion analysis in order to find the regulatory regions responsible for bidirectional expression activity (Figure 1). The specific primers used for PCR amplification to generate different 5′ and 3′ truncated fragments are shown in Table 2. Vector construction, callus culture and transformation, histochemical and fluorometric analysis of GUS activity, histological and quantitative analysis of GFP were performed as described above.

### Conservation analysis of the four bidirectional promoters and bioinformatic identification of their conserved sequences

The conserved arrangements of the four gene pairs in six gramineous plants (*O. sativa, Sorghum bicolor, Setaria italica, Brachypodium distachyon, Zea mays*, and *Triticum aestivum*) were identified with the information from the Ensembl Plants database (http://plants.ensembl.org/index.html). The bidirectional genes whose homologous genes in other species were still arranged in a bidirectional architecture were considered to be regulated by conserved bidirectional promoters (c-BIP); otherwise, they were considered to be regulated by non-conserved bidirectional promoters (n-BIP).

The conserved sequences in the four bidirectional promoters were discovered by MEME (http://meme-suite.org//tools/meme) and their frequencies in the intergenic regions between divergent gene pairs in rice genome were identified by FIMO (*p* < 1E-8) using the reference of random promoters (http://meme-suite.org//tools/fimo).

## Results

### Selection of four novel bidirectional promoters in rice genome and their functional characterization in transgenic plants

Based on RNA-seq and microarray data, we chose four divergent gene pairs (Table 1) according to the criteria in Section Methods and isolated their intergenic regions for functional identification. The four fragments were respectively cloned to a dual reporter vector pDX2181 and transformed into rice variety Zhonghua 11.

**Figure 2:**
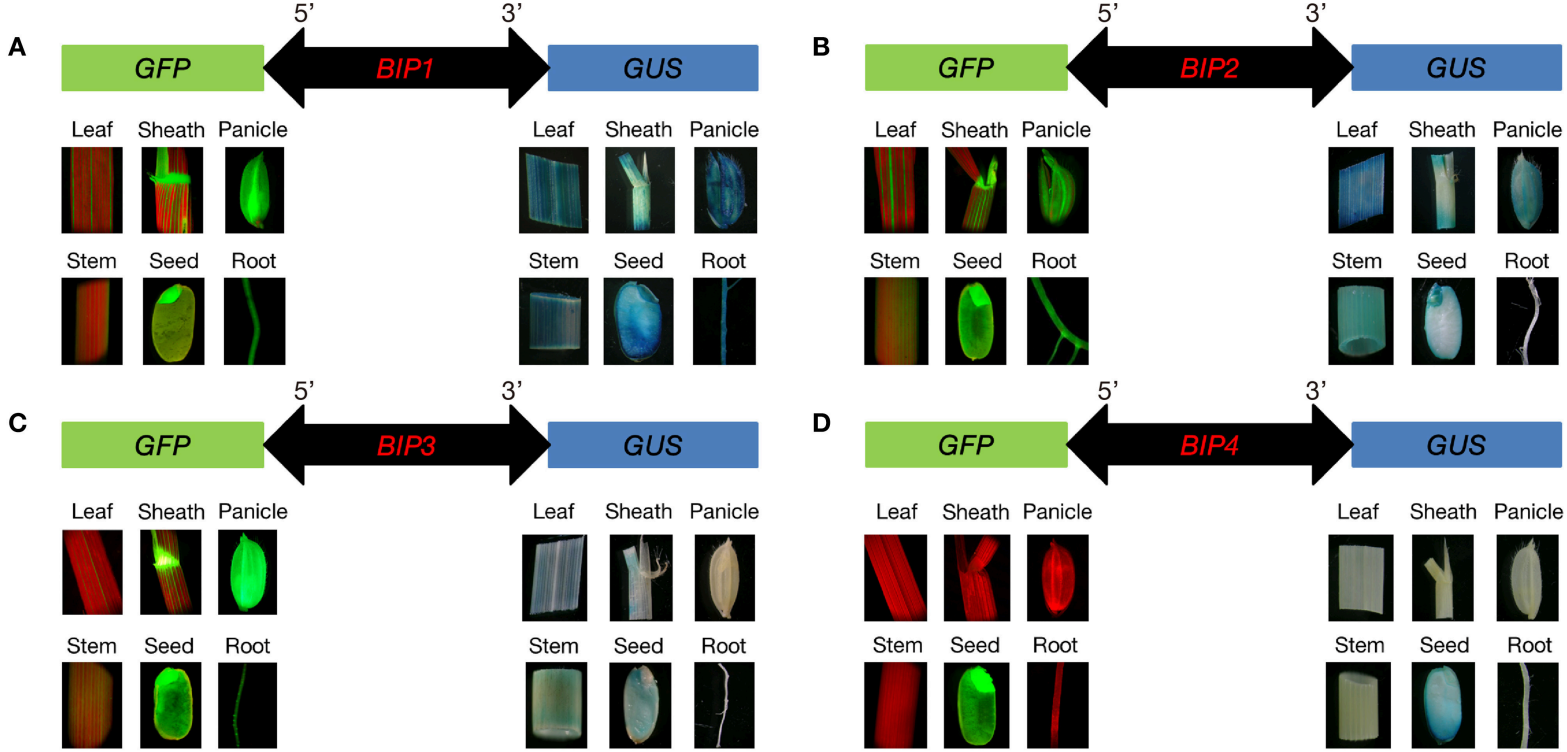
**Histological analysis of *GFP* and *GUS* expression in various tissues of the transgenic plants containing different *GFP*/bidirectional promoter/*GUS* fusions. (A–D)**, plants containing *GFP::BIP1*-*BIP4::GUS*. Localization of GFP is shown at the left area; Localization of GUS is shown at the right area.

**Figure 3:**
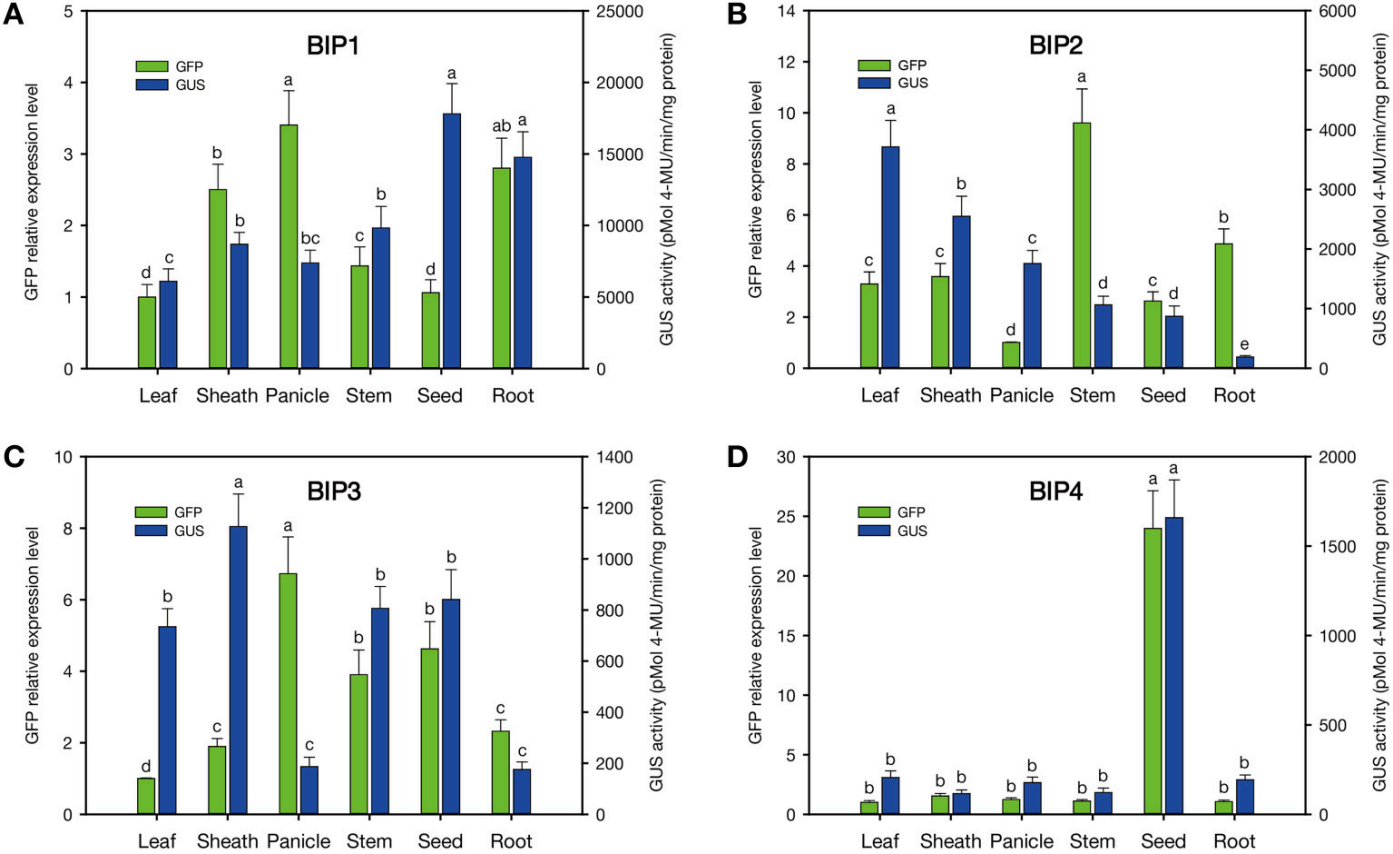
**Quantitative analysis of GFP and GUS expression in various tissues of the transgenic plants containing different *GFP*/bidirectional promoter/*GUS* fusions. (A–D)**, plants containing *GFP::BIP1*-*BIP4::GUS*. a, b, c, d, e: significant difference (*P* < 0.05). Error bars indicate _SE_ based on five independent biological replicates.

According to the results of GUS and GFP assays of the transgenic plants, all the intergenic regions showed bidirectional expression activity in various tissues. Histological GUS and GFP analysis of the transgenic plants showed that four novel bidirectional promoters (*BIP1, BIP2, BIP3*, and *BIP4*) were successfully identified in our work. Among them, *BIP1, BIP2*, and *BIP3* showed bidirectional constitutive expression patterns and *BIP4* showed bidirectional seed-specific expression pattern (Figure 2). Analysis of GUS fluorometric activities in various tissues of *BIP1* transgenic plants (Figure 3) showed that the expression efficiency of *BIP1* toward 3′ was the highest in the seed, which showed a GUS enzymatic activity of 17806 ± 2108 pmol 4-MU/min/mg protein, followed by in the root, which exhibited a GUS enzymatic activity of 14769 ± 1782 pmol 4-MU/min/mg protein, while the GUS enzymatic activities in the stem, sheath, panicle, and leaf were 9825 ± 1510, 8681 ± 834, 7380 ± 895, and 6092 ± 875 pmol 4-MU/min/mg protein, respectively. Analysis of GFP expression in *BIP1* transgenic plants (Figure 3) revealed that the expression efficiency of *BIP1* toward 5′ was the highest in the panicle, which was 2.4-fold higher than that in the leaf. While the expression levels of GFP in the root, sheath, stem, and seed were 1.8-, 1.5-, 0.4-, and 0.1-fold higher than that in the leaf, respectively. GUS assays of *BIP2* transgenic plants (Figure 3) indicated that the expression efficiency of *BIP2* toward 3′ was the highest in the leaf, which showed a GUS enzymatic activity of 3713 ± 445 pmol 4-MU/min/mg protein, and the GUS activities in the sheath, panicle, stem, and seed were 2548 ± 339, 1755 ± 220, 1063 ± 145, and 872 ± 173 pmol 4-MU/min/mg protein, respectively, while GUS activity was hardly detected in the root. Analysis of GFP expression in *BIP2* transgenic plants (Figure 3) showed that the expression efficiency of *BIP2* toward 5′ was the highest in the stem, which was 8.6-fold higher than that in the panicle, while the expression levels of GFP in the root, sheath, leaf, and seed were 3.9-, 2.6-, 2.3-, and 1.6-fold higher than that in the panicle, respectively. *BIP3* transgenic plants showed GUS enzymatic activities of 1126 ± 128, 841 ± 117, 806 ± 85, and 734 ± 71 pmol 4-MU/min/mg protein in the sheath, seed, stem, and leaf, respectively, while GUS activity was hardly detected in the panicle and root. The expression efficiency of *BIP3* toward 5′ was the highest in the panicle, which was 5.7-fold higher than that in the leaf, while the expression levels of GFP in the seed, stem, root, and sheath were 3.6-, 2.9-, 1.3-, and 0.9-fold higher than that in the leaf, respectively. *BIP4* showed a bidirectional seed-specific expression pattern, as a GUS enzymatic activity of 1659 ± 211 pmol 4-MU/min/mg protein was detected in the seed while almost no GUS activity was detected in other tissues, and a 23-fold higher expression level of GFP was observed in the seed compared with in the leaf. In addition, these results indicate that all the bidirectional promoters identified here direct gene expression in an orientation-independent manner; namely, the expression patterns in opposite directions of these bidirectional promoters are similar, which is consistent with the co-expression characteristics of the adjacent genes.

**Figure 4:**
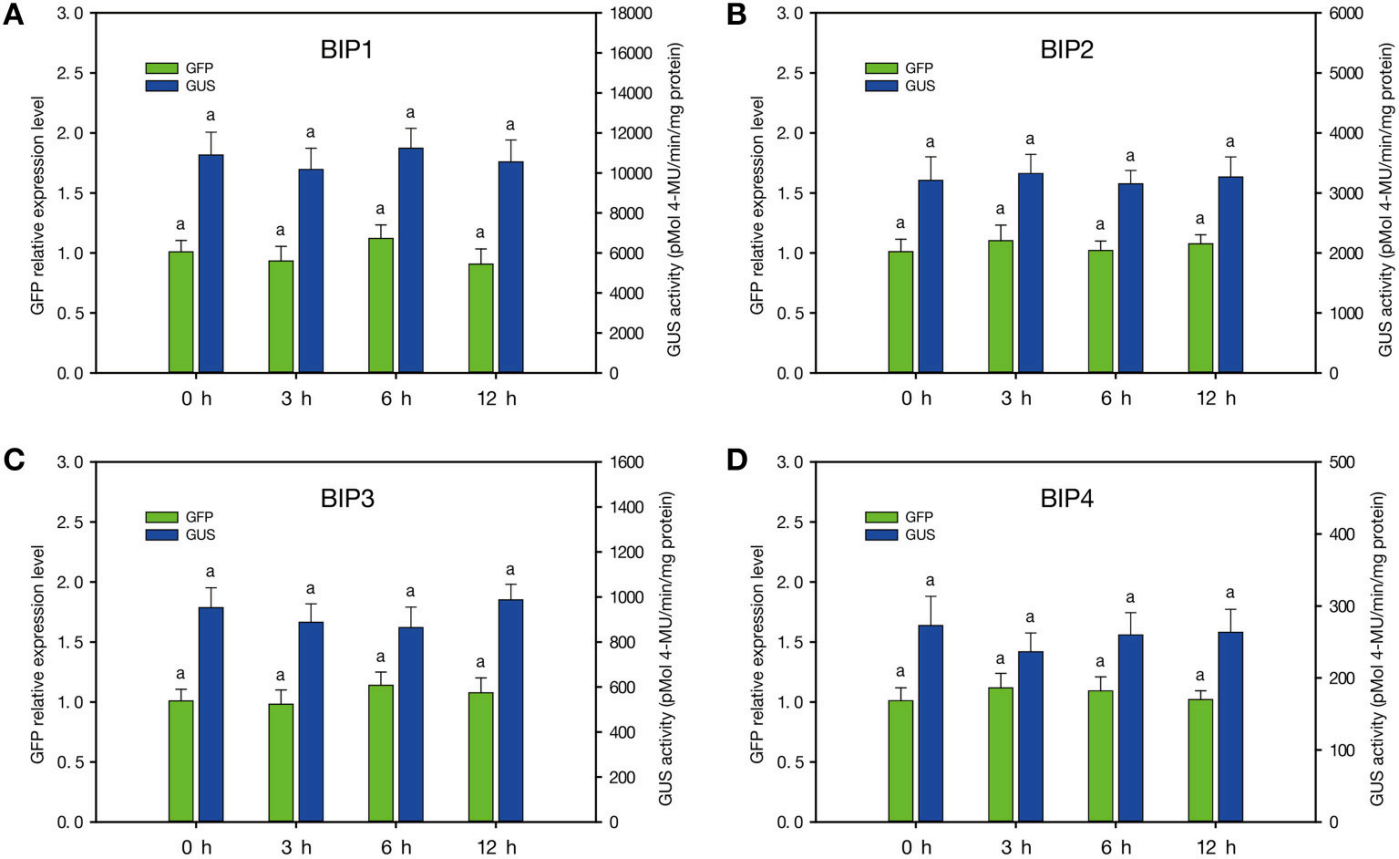
**Quantitative analysis of GFP and GUS expression of the *BIPs* transgenic plants in response to melatonin**. **(A–D)**, plants containing *GFP::BIP1*-*BIP4::GUS*. a: no significant difference. Error bars indicate _SE_ based on five independent biological replicates.

Melatonin is one of the most important hormones in plant and animal. In order to test the response of the four bidirectional promoters to melatonin, melatonin treatment was performed on the *BIPs* transgenic plants. The results of GUS and GFP assays indicated that the four bidirectional promoters were not induced by melatonin (Figure 4). Hence, it can be inferred that no melatonin-responsive *cis*-element was harbored in these promoters.

### Identification of the expression regulatory regions in *BIP1*

**Figure 5:**
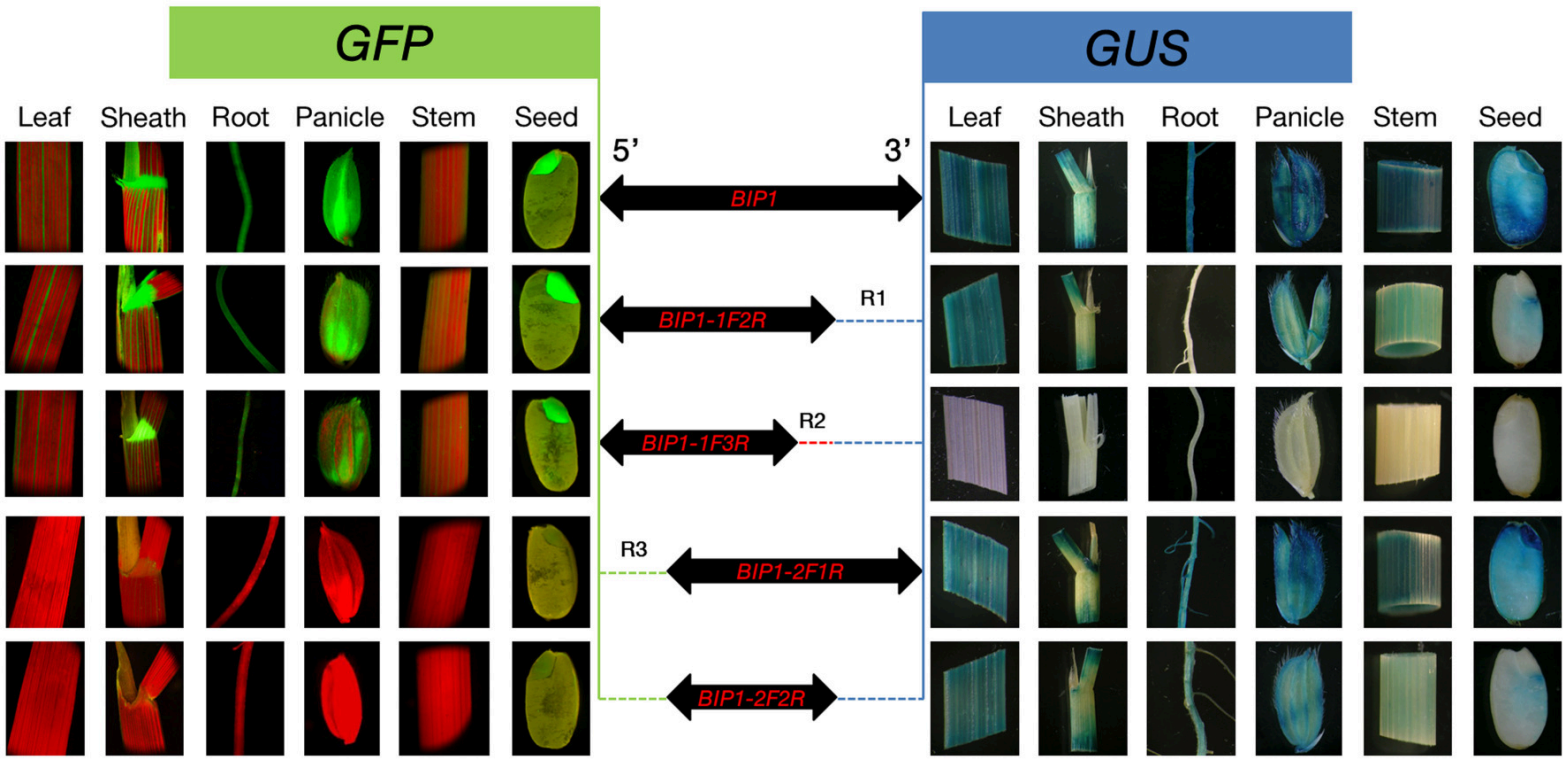
**Histological analysis of *GFP* and *GUS* expression in various tissues of the transgenic plants containing different *GFP*/*BIP1* deleted version/*GUS* fusions**. Localization of GFP is shown at the left area; Localization of GUS is shown at the right area. R1 (blue dashed line), region 1; R2 (red dashed line), region 2; R3 (green dashed line), region 3.

**Figure 6:**
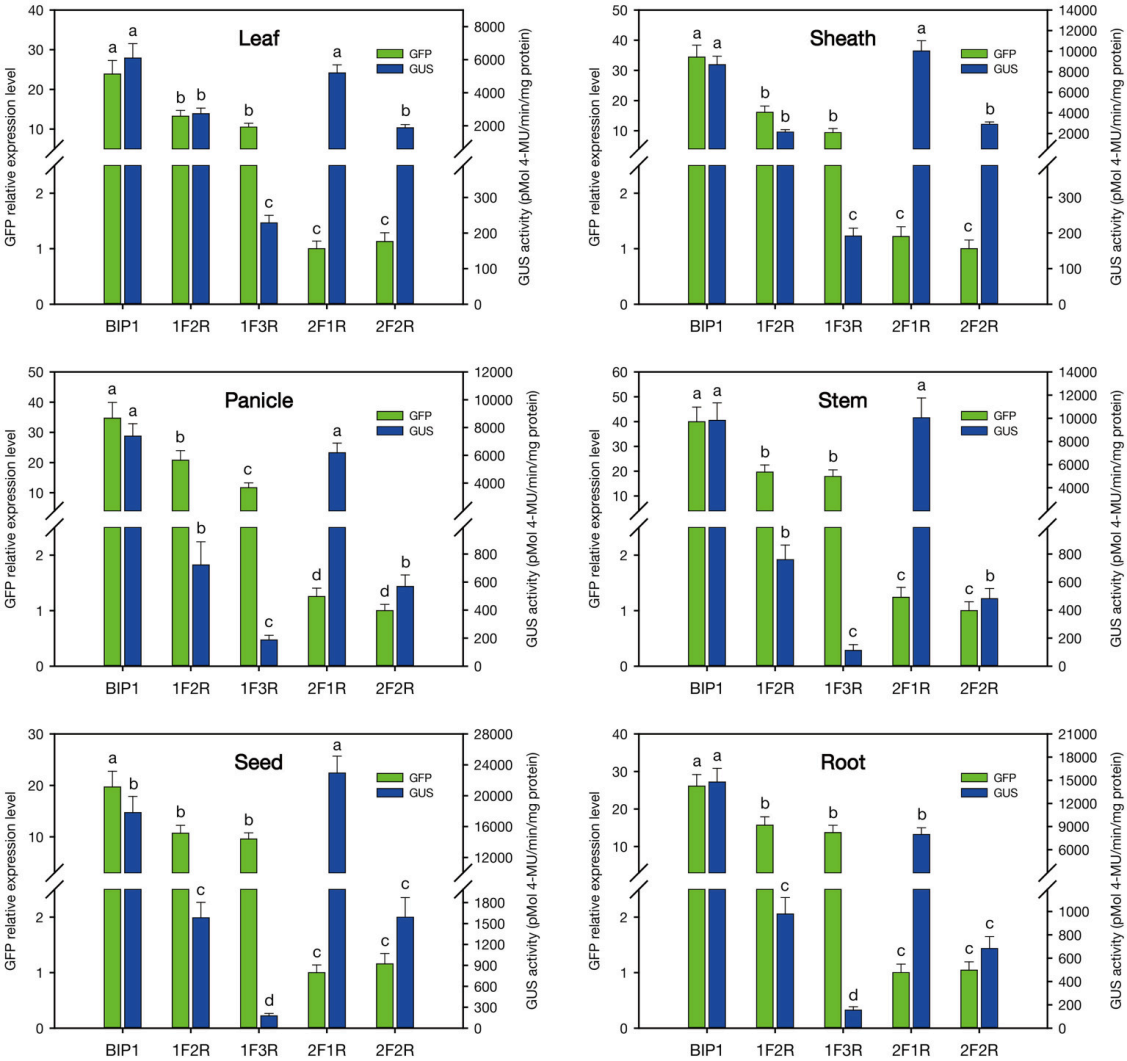
**Quantitative analysis of GFP and GUS expression in various tissues of the transgenic plants containing different *GFP*/*BIP1* deleted version/*GUS* fusions**. 1F2R, plants containing *GFP::BIP1*-*1F2R::GUS*; 1F3R, 2F1R and 2F2R follow the same pattern. a, b, c, d: significant difference (*P* < 0.05). Error bars indicate _SE_ based on five independent biological replicates.

In order to identify the regulatory regions in *BIP1*, 5′ and 3′ deletion analysis of this promoter was performed. A series of truncated *BIP1* were respectively cloned to pDX2181 and transformed into Zhonghua 11. Transgenic plants carrying *BIP1-1F2R* showed much lower GUS activity than *BIP1* transgenic plants in various tissues, especially in the root, stem, seed and panicle, whose GUS activities were lower than 10% of that in the corresponding tissues of *BIP1* transgenic plants (Figures 5, 6). It thus could be inferred that region 1 could greatly increase the transcriptional activity of *BIP1* toward 3′. Meanwhile, *BIP1-1F2R* transgenic plants also showed an obvious decrease of GFP expression in various tissues compared with *BIP1* transgenic plants (Figure 6). These results could be integrated to reveal that region 1 is a bidirectional transcription-enhancing region of *BIP1*. Further truncating in 3′ of *BIP1* led to complete abolishment of GUS activity in *BIP1-1F3R* transgenic plants, while the GFP expression level was not obviously reduced in *BIP1-1F3R* transgenic plants compared with in *BIP1-1F2R* transgenic plants. These results suggest that region 2 is the essential region responsible for the basic expression activity of 3′ but not for that of 5′. Transgenic plants carrying *BIP1-2F1R* or *BIP1-2F2R* showed no expression of GFP, indicating that truncating 5′ of *BIP1* will completely abolish 5′ expression activity of the promoter. GUS assays in *BIP1-2F1R* transgenic plants revealed that truncating 5′ of *BIP1* caused slight changes of 3′ expression activity in most tissues except for the root, which showed obviously decreased GUS activity compared with that of *BIP1* transgenic plants (Figure 6). These results suggest that region 3 is the essential region responsible for the basic expression activity of 5′ but not for that of 3′; however, it can positively regulate the expression activity of 3′ in the root.

### Conserved arrangement of the four gene pairs regulated by bidirectional promoters

The sequences of promoters are known to be variable (Müller et al., 2007). Therefore, in order to analyze the conservation of the four bidirectional promoters in different species, we investigated the conservation of the four gene pairs regulated by these promoters.

The conserved arrangement of the four gene pairs in six gramineous plants was identified with information from the Ensembl Plants database. The bidirectional genes whose homologous genes in other species were still arranged in a bidirectional architecture were considered to be regulated by c-BIP; otherwise, they were considered to be regulated by n-BIP (Table 3). It was found that *BIP1* and *BIP3* were the most c-BIP in the six gramineous plants. *BIP1* was conserved in *O. sativa, S. bicolor, B. distachyon*, and *Z. mays*; *BIP3* was conserved in *O. sativa, S. bicolor, S. italic*, and *B. distachyon*; *BIP4* was conserved in *O. sativa, S. bicolor*, and *S. italica*; while *BIP2* was only conserved in *O. sativa* and *T. aestivum*.

**Table 3:** **Conservation analysis of the four bidirectional promoters in gramineous plants**.

	***Oryza sativa***	***Sorghum bicolor***	***Setaria italica***	***Brachypodium distachyon***	***Zea mays***	***Triticum aestivum***
*BIP1*	C/C, c-BIP	C/C, c-BIP	C/C, n-BIP	C/C, c-BIP	C/C, c-BIP	C/C, n-BIP
*BIP2*	C/C, c-BIP	C/C, n-BIP	C/C, n-BIP	C/C, n-BIP	C/C, n-BIP	C/C, c-BIP
*BIP3*	C/C, c-BIP	C/C, c-BIP	C/C, c-BIP	C/C, c-BIP	C/C, n-BIP	C/C, n-BIP
*BIP4*	C/C, c-BIP	C/C, c-BIP	C/C, c-BIP	C/C, n-BIP	C/C, n-BIP	C/C, n-BIP

*If 5′ gene/3′ gene of the bidirectional promoters had homologous gene in another species (C/C) and the homologous genes were still arranged in bidirectional architecture, they were considered to be regulated by conserved bidirectional promoters (c-BIP). Otherwise, they were considered to be regulated by non-conserved bidirectional promoters (n-BIP)*.

### Potential *cis*-sequences involved in bidirectional expression

**Figure 7:**
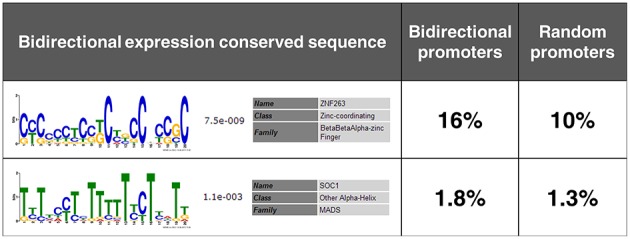
**Two novel *cis*-sequences conserved in the four identified bidirectional promoters**. The frequencies of the two *cis*-sequences in the intergenic regions between divergent gene pairs in rice genome and in the random promoters used as a reference set are shown on the second column and the third column, respectively.

By MEME, two conserved *cis*-sequences in the four bidirectional promoters were identified (Figure 7). *Cis*-sequence 1 was a G/C-rich sequence, while *cis*-sequence 2 was an A/T-rich sequence. Subsequently, the frequencies of these *cis*-sequences in the intergenic regions between divergent gene pairs in rice genome were analyzed by FIMO using the reference of random promoters. Consistent with the expectation, the two *cis*-sequences conserved in the four bidirectional promoters both showed overrepresentation in potential bidirectional promoters in rice genome compared with random promoters. This result further reveals that the two novel *cis*-sequences are probably involved in bidirectional expression.

## Discussion

In this study, we initiatively combined RNA-seq data and cDNA microarray data to discover potential bidirectional promoters in rice. Four adjacent and oppositely transcribed gene pairs were selected based on their expression levels and correlations. The intergenic regions between the four gene pairs were successfully cloned, and all of them were identified to be bidirectional promoters, confirming the feasibility of our method for discovering bidirectional promoters. This is the first study to clone and identify bidirectional promoters using two reporter genes simultaneously with stable transformation in rice. Among the four identified bidirectional promoters, *BIP1* shows high expression efficiency in various tissues, and thus has a high application potential in genetic engineering, such as driving two resistant genes simultaneously in transgenic breeding against pest/disease stress, which can confer more strong, broad, and durable resistance in rice (Du et al., 2009; Shah et al., 2009; Yang et al., 2011; Wang et al., 2015a). Rice is one of the most important food crops in the world, and its seed is the edible part consumed by human. Therefore, it is highly necessary to improve the nutrient quality of the seed (Ha et al., 2010; Li Y. et al., 2011, 2014; Ogo et al., 2013). Efficient and specific expression of multiple target genes for seed improvement in rice could hardly be realized without seed-specific promoter. In this work, *BIP4* shows a bidirectional seed-specific expression pattern, indicating its high application potential in the improvement of seed quality by specifically driving multiple genes. The results of 5′ and 3′ deletion analysis reveal that region 1 is the bidirectional transcription-enhancing region of *BIP1*; region 2 is the essential region specifically responsible for the basic expression activity of 3′; region 3 is the essential region responsible for the basic expression activity of 5′ but not for that of 3′, while it can positively regulate the 3′ expression activity in the root.

Conservation analysis of the four bidirectional promoters in gramineous plants reveals the possible co-evolution of adjacent genes regulated by these promoters. The bidirectional arrangement of LOC_Os02g42314 and LOC_Os02g42320, which are regulated by *BIP1*, is conserved in four species of six gramineous plants, suggesting that they are relatively conserved during co-evolution. The functional annotations of these two genes, which are “ubiquitin-conjugating enzyme” and “peptidase,” show that both of them are structural genes conserved during evolution. Moreover, the functional relationship between the two genes further supports their co-evolutionary conservation. LOC_Os02g47000 and LOC_Os02g47010, which are regulated by *BIP3*, also show conserved arrangement in four species of six gramineous plants. Although the functional annotation of LOC_Os02g47000 is unclear, considering that LOC_Os02g47010 is annotated to encode “secretory carrier-associated membrane protein,” we speculate that LOC_Os02g47000 may encode a structural protein related to secretory pathway.

So far, many *cis*-regulatory sequences have been identified, which are involved in inducible expression (Liu et al., 2010, 2014; Yuan et al., 2011; Koschmann et al., 2012; Walcher and Nemhauser, 2012) and tissue-specific expression (Hartmann et al., 2005; Cai et al., 2007; Ye et al., 2012; Wang et al., 2015b). A previous report also has suggested that several known *cis*-sequences might be related to bidirectional expression (Dhadi et al., 2009). Here, we used the experimentally verified bidirectional promoters to predict two *cis*-sequences related to bidirectional expression which had not been identified in rice genome. Subsequently, overrepresentation of the two novel *cis*-sequences in the intergenic regions between divergent gene pairs further reveals their involvement in bidirectional expression. Interestingly, *cis*-sequence 1 is a G/C-rich sequence, which is consistent with the characteristics of higher GC content in bidirectional promoters; however, *cis*-sequence 2 is an A/T-rich sequence, which might be a new finding in the sequence characteristics of bidirectional promoters. Overall, the novel bidirectional promoters identified using two reporter genes simultaneously with stable transformation in rice are expected to have high applicability in genetic engineering. Our study proposes a feasible method for selecting, cloning, and functionally identifying bidirectional promoters as well as for discovering their bidirectional regulatory regions and conserved sequences in rice.

## Author contributions

YL and RW conceived and designed the experiments. RW, YY, MZ, and MY performed the experiments. RW and YY performed the data analysis. RW and YL wrote the paper. FZ and HC revised the paper. YL secured the funds to support this research.

### Conflict of interest statement

The authors declare that the research was conducted in the absence of any commercial or financial relationships that could be construed as a potential conflict of interest.
